# Fabrication and characterization of carbon-based counter electrodes prepared by electrophoretic deposition for dye-sensitized solar cells

**DOI:** 10.1186/1556-276X-7-53

**Published:** 2012-01-05

**Authors:** Hyunkook Kim, Hyonkwang Choi, Sookhyun Hwang, Youngjoo Kim, Minhyon Jeon

**Affiliations:** 1 Department of Nano Systems Engineering, Center for Nano Manufacturing, Inje University, Gimhae, Gyungnam, 621-749, Republic of Korea

**Keywords:** dye-sensitized solar cells, counter electrodes, graphene, single-walled carbon nanotubes, electrophoretic deposition.

## Abstract

Three different carbon-based counter electrodes are investigated in light of catalytic activities such as electrochemical frequencies and interface impedances. We fabricated carbon-based counter electrodes of dye-sensitized solar cells [DSSCs] using graphene, single-walled carbon nanotubes [SWNTs], and graphene-SWNT composites by electrophoretic deposition method. We observed the optical and electrochemical properties of the carbon-based counter electrodes. The DSSC with the graphene-deposited counter electrode demonstrated the best conversion efficiency of 5.87% under AM 1.5 and 1 sun condition. It could be utilized for a low-cost and high-throughput process for DSSCs.

## Introduction

Dye-sensitized solar cells [DSSCs] have emerged as the next generation of photovoltaic devices, offering several advantages, including moderate light-to-electricity conversion efficiency, easy fabrication, and low cost [1-4]. Generally, a DSSC is composed of a mesoporous nanocrystalline film (normally titanium oxide), to whose surface is attached a monolayer of the charge-transfer dye molecule, an electrolyte containing a dissolved iodide/tri-iodide redox couple, and a counter electrode. The role of counter electrodes is to transfer electrons from the external circuit to the tri-iodide and iodine in the redox electrolyte [5]. Most commonly, Pt counter electrodes are utilized; however, despite their excellent properties, they suffer from several limitations, e.g., difficulty in large-scale production and high economic cost. Carbon nanomaterials provide a promising alternative to Pt owing to their intrinsic attractive features, notably their high electrical conductivity, corrosion resistance, and excellent electrocatalytic activity, as well as their increasingly affordable cost.

The application of various carbon nanomaterials, such as carbon blacks, carbon nanotubes, and graphenes, to counter electrodes has been widely documented in the literature [6-12]. We reported that chemically converted graphene-based carbon nanocomposites and chemical-vapor-deposited graphene-based carbon nanocomposites had energy conversion efficiencies of 3.0% and 4.46%, respectively. However, several difficulties such as low cost and mass production process have hampered the realization of these materials as a counter electrode for DSSCs [13,14].

In order to overcome those problems, we investigated counter electrodes fabricated with three different carbon-based materials such as graphene, single-walled carbon nanotubes [SWNTs], and graphene-SWNT composites using electrophoretic deposition [EPD]. The EPD method is an automated and high-throughput process that has been widely employed in the industry; it can provide a homogeneous and robust film on the surface of the substrate [15-17]. Herein, we present fabrication and characterization results of counter electrodes of graphene, SWNTs, and graphene-SWNT composites by the EPD method using a dispersion solution of CNTs and graphene.

## Experimental details

Graphenes were produced from graphite oxides, which were synthesized using a modified Hummers' method [18-20]. SWNTs were purchased from Hanwha Nanotech Corporation (Incheon, South Korea), which had a diameter of 1.5 to 3 nm and a length of a few micrometers. Subsequently, an EPD solution was prepared to deposit the graphenes, SWNTs, and carbon composites on fluorine-doped tin oxide [FTO] substrates. Chemically converted graphenes, SWNTs, magnesium nitrate, and ethanol were mixed together in an ultrasonicator for several hours. The FTO glass (7 Ω·cm^-2^) and a stainless steel substrate were then immersed in the EPD solution. The distance between the FTO and the stainless steel substrate was kept at 1 cm, and a voltage of 30 V was applied. The counter electrodes were annealed at 600°C for 1 min, after which they were gradually cooled under nitrogen gas at ambient temperature.

A porous TiO_2 _film was coated onto the FTO glass using the doctor-blade method; the fabrication was then sintered at 450°C for 1 h, which resulted in a film thickness of approximately 30 μm. The mesoporous TiO_2 _film was then immersed in a solution of the N-719 dye (Ruthenizer 535-bisTBA, Solaronix, Aubonne, Switzerland) with a concentration of 0.5 mmol/L in ethanol for a period of 36 h at room temperature. After that time, the TiO_2 _electrode and counter electrode were sandwiched with an approximately 60-μm-thick (before melting) surlyn polymer foil as a spacer and sealed by keeping the cell in a hot-press at 110°C for 10 s. The liquid electrolyte (AN-50, Solaronix) was injected through predrilled holes on the counter electrode, which were next sealed by the surlyn polymer foil and a cover glass.

The deposited SWNTs, graphenes, and carbon composites were characterized by field-emission scanning electron microscopy [FE-SEM] and ultraviolet-visible spectroscopy. The cells were illuminated using a solar simulator (PEC-L01, Peccell Technologies, Inc., Yokohama, Kanagawa, Japan) under AM 1.5 (100 mW/cm^2^) irradiation. The energy conversion efficiency of the cells was recorded by an electrochemical impedance analyzer (Compacstat, Ivium Technologies, Fernandina Beach, FL, USA). Electrochemical impedance spectroscopy measurements were carried out with a bias illumination of 100 mW/cm^2 ^under an open-circuit condition and in a frequency range of 0.1 Hz to 100 KHz.

## Results and discussion

**Figure 1:**
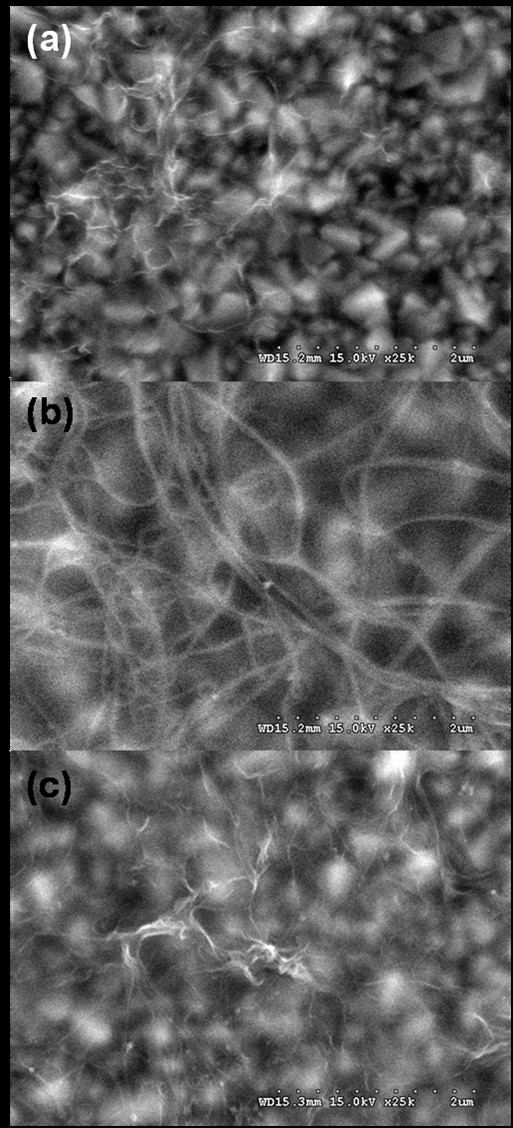
**FE-SEM images**. (**a**) Graphene-deposited FTO substrate. (**b**) SWNT-deposited FTO substrate. (**c**) Graphene-SWNT composite-deposited FTO substrate.

Figure 1 shows the FE-SEM images of deposited (a) graphenes, (b) SWNTs, and (c) graphene-SWNT composites on the FTO substrates. Deposited graphenes (a) were identified by their different contrasts, and they showed the presence of graphene wrinkles formed during the EPD deposition. In the case of the SWNT electrode (b), relatively thick SWNT layers were deposited onto the substrates. Finally, the deposited graphene-SWNT composite electrode (c) showed the simultaneous presence of graphene wrinkles and SWNTs.

**Figure 2:**
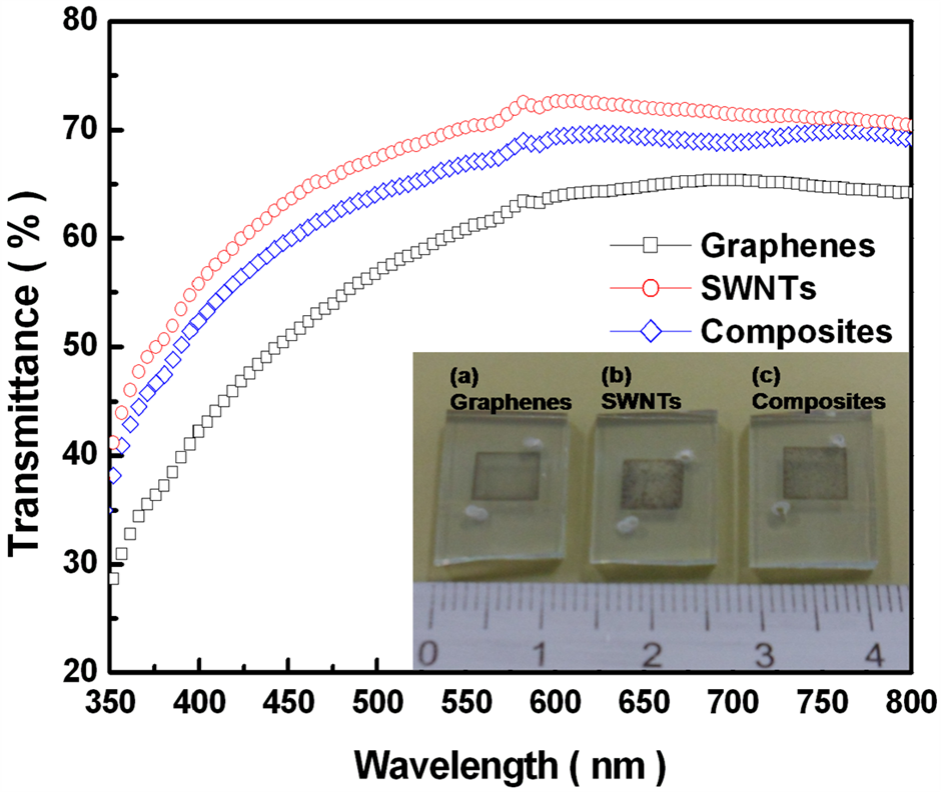
**Transmittance spectra of carbon-based counter electrodes**. The inset shows different deposition materials: (**a**) graphenes, (**b**) SWNTs, and (**c**) graphene-SWNT composites.

The optical transmittance of the graphene, SWNT, and carbon composite electrodes was then measured to investigate their potential for use as transparent counter electrodes (Figure 2). The inset shows a photograph of each counter electrode. In the visible range (at 550 nm), transmittances of the graphene, SWNTs, and graphene-SWNT composite electrodes were measured to be 62%, 70%, and 67%, respectively.

**Figure 3:**
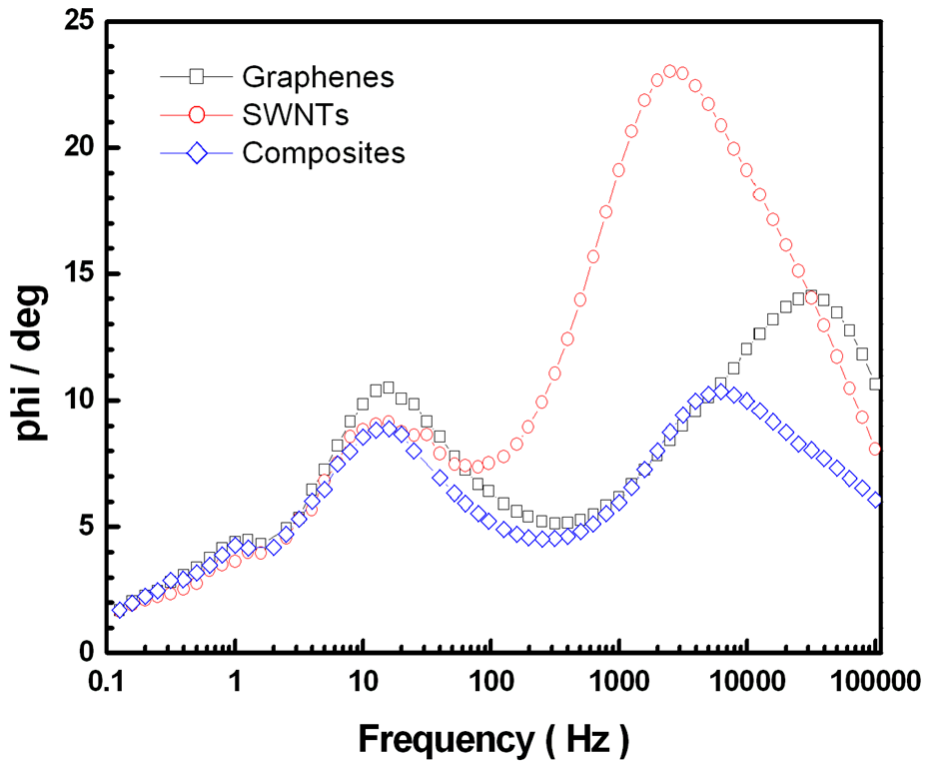
**Bode phase plots of DSSCs**. Bode phase plots of DSSCs with different counter electrodes: graphenes (square), SWNTs (circle), and graphene-SWNT composites (diamond).

Subsequently, DSSCs were fabricated using counter electrodes with three different carbon-based materials with the objective of evaluating the electrochemical properties of the counter electrodes and the energy conversion efficiencies of cells. Figure 3 shows the Bode phase plots of the DSSCs with graphenes, SWNTs, and graphene-SWNT composite counter electrodes. Since the frequency peak in the high-frequency region in the Bode phase plot is related to the charge transfer at the interfaces of electrolyte/counter electrodes, we only focus on the characteristic peaks in this region. As can be seen from the figure, redox frequencies on the graphene, carbon nanocomposite, and SWNT counter electrodes were measured to be 31.6, 6.3, and 2.5 KHz, respectively.

**Figure 4:**
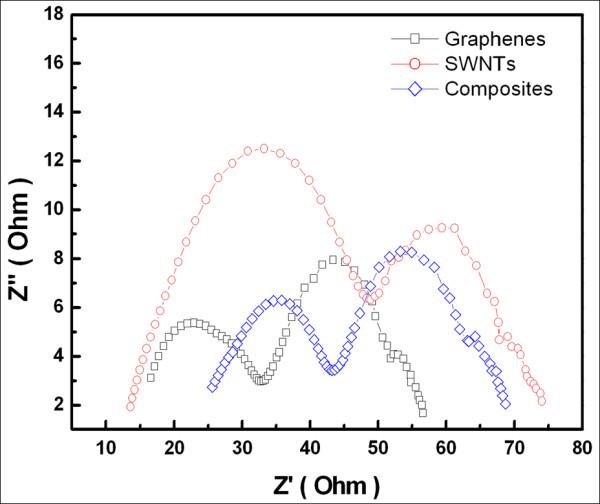
**Nyquist plot of DSSCs**. Nyquist plot of DSSCs with different counter electrodes: graphenes (square), SWNTs (circle), and graphene-SWNT composites (diamond).

The Nyquist plots of those three counter electrodes are shown in Figure 4. A Nyquist plot typically contains two or three semicircles: the first circle in the high-frequency range is related to the interface between the electrolyte and the counter electrode, whereas the second circle is related to the TiO_2_/electrolyte interface. As shown in the figure, the resistances (*R*_ct1_) between the electrolyte and the graphenes, SWNTs, and carbon nanocomposite counter electrodes of the DSSC were measured at 16.2, 35.3, and 17.6 Ω, respectively.

**Figure 5:**
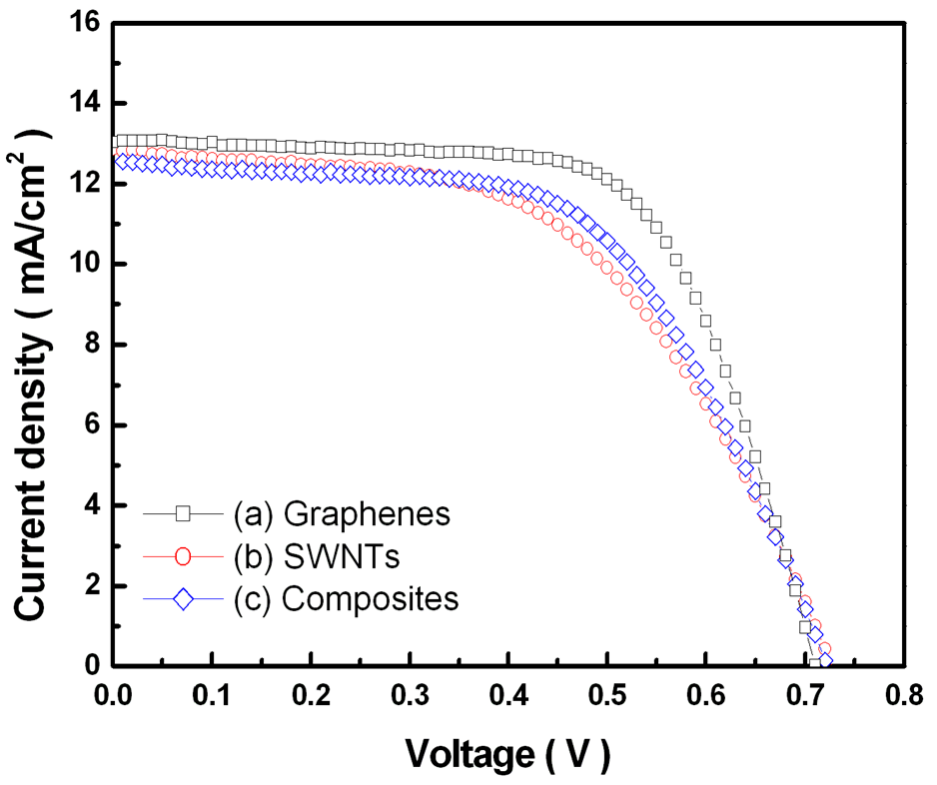
***J*-*V *characteristics of DSSCs with different counter electrodes**. (**a**) Graphenes. (**b**) SWNTs. (**c**) Graphene-SWNT composites.

Figure 5 shows the current density-voltage characteristics of the DSSCs with carbon nanomaterials. The redox frequency [*R*_ct1_], open-circuit voltage [*V*_oc_], short-circuit photocurrent density [*J*_sc_], fill factor [FF], and energy conversion efficiency [*η*] are listed in Table 1. From the values listed in the table, it can be said that graphene is the most suitable material for a counter electrode, followed by carbon nanocomposites and SWNTs.

**Table 1:** Experimental data of DSSCs with counter electrodes of differential carbon-based materials

	***R***_**ct1**_ (Hz)	***R***_**ct1**_ (Ω)	***V***_**oc**_ (V)	***J***_**sc**_ **(mA/cm**^**2**^**)**	FF (%)	*η* (%)
Graphenes	31, 600	16.212	0.7	13.1	63.6	5.87
SWNTs	2, 510	35.347	0.71	13.0	52.3	4.94
Composites	6, 310	17.631	0.7	12.7	56.5	5.17

*R*_ct1_, redox frequency; *V*_oc_, open-circuit voltage; *J*_sc_, short-circuit photocurrent density; FF, fill factor; *η*, energy conversion efficiency; SWNTs, single-walled carbon nanotubes.

## Conclusion

In this report, we demonstrated the fabrication of carbon nanomaterials deposited on FTO substrates by the EPD method and their application as counter electrodes for DSSCs. Our results provided evidence that graphene, SWNTs, and graphene-SWNT composites could perform sufficiently well as counter electrodes for DSSCs. Comparison of the *η *and FF of the counter electrodes with three different carbon-based materials measured under similar deposition conditions of optical transmittance showed that graphene is the most suitable material for application as a counter electrode in DSSCs among them. Based on this finding, in the future, we intend to conduct further studies for improving the performance of graphene-based counter electrodes in order to realize DSSCs with higher efficiency.

## Competing interests

The authors declare that they have no competing interests.

## Authors' contributions

HK fabricated the cells and wrote the paper. HK and HC did the characterization and imaging of the solar cells. SH and YK helped design the experimental study and advised on the project. MJ developed the conceptual framework and supervised the work. All authors read and approved the final manuscript.
